# RIFLE: a Phase II trial of stereotactic ablative radiotherapy combined with fruquintinib and tislelizumab in metastatic colorectal cancer

**DOI:** 10.1093/gastro/goad063

**Published:** 2023-10-11

**Authors:** Kun Wang, Yajie Chen, Zhiyuan Zhang, Ruiyan Wu, Menglong Zhou, Wang Yang, Juefeng Wan, Lijun Shen, Hui Zhang, Yan Wang, Xu Han, Jiazhou Wang, Zhen Zhang, Fan Xia

**Affiliations:** Department of Radiation Oncology, Fudan University Shanghai Cancer Center, Shanghai, P. R. China; Department of Oncology, Shanghai Medical College, Fudan University, Shanghai, P. R. China; Shanghai Key Laboratory of Radiation Oncology, Shanghai, P. R. China; Department of Radiation Oncology, Fudan University Shanghai Cancer Center, Shanghai, P. R. China; Department of Oncology, Shanghai Medical College, Fudan University, Shanghai, P. R. China; Shanghai Key Laboratory of Radiation Oncology, Shanghai, P. R. China; Department of Radiation Oncology, Fudan University Shanghai Cancer Center, Shanghai, P. R. China; Department of Oncology, Shanghai Medical College, Fudan University, Shanghai, P. R. China; Shanghai Key Laboratory of Radiation Oncology, Shanghai, P. R. China; Department of Radiation Oncology, Fudan University Shanghai Cancer Center, Shanghai, P. R. China; Department of Oncology, Shanghai Medical College, Fudan University, Shanghai, P. R. China; Shanghai Key Laboratory of Radiation Oncology, Shanghai, P. R. China; Department of Radiation Oncology, Fudan University Shanghai Cancer Center, Shanghai, P. R. China; Department of Oncology, Shanghai Medical College, Fudan University, Shanghai, P. R. China; Shanghai Key Laboratory of Radiation Oncology, Shanghai, P. R. China; Department of Radiation Oncology, Fudan University Shanghai Cancer Center, Shanghai, P. R. China; Department of Oncology, Shanghai Medical College, Fudan University, Shanghai, P. R. China; Shanghai Key Laboratory of Radiation Oncology, Shanghai, P. R. China; Department of Radiation Oncology, Fudan University Shanghai Cancer Center, Shanghai, P. R. China; Department of Oncology, Shanghai Medical College, Fudan University, Shanghai, P. R. China; Shanghai Key Laboratory of Radiation Oncology, Shanghai, P. R. China; Department of Radiation Oncology, Fudan University Shanghai Cancer Center, Shanghai, P. R. China; Department of Oncology, Shanghai Medical College, Fudan University, Shanghai, P. R. China; Shanghai Key Laboratory of Radiation Oncology, Shanghai, P. R. China; Department of Radiation Oncology, Fudan University Shanghai Cancer Center, Shanghai, P. R. China; Department of Oncology, Shanghai Medical College, Fudan University, Shanghai, P. R. China; Shanghai Key Laboratory of Radiation Oncology, Shanghai, P. R. China; Department of Radiation Oncology, Fudan University Shanghai Cancer Center, Shanghai, P. R. China; Department of Oncology, Shanghai Medical College, Fudan University, Shanghai, P. R. China; Shanghai Key Laboratory of Radiation Oncology, Shanghai, P. R. China; Department of Radiation Oncology, Fudan University Shanghai Cancer Center, Shanghai, P. R. China; Department of Oncology, Shanghai Medical College, Fudan University, Shanghai, P. R. China; Shanghai Key Laboratory of Radiation Oncology, Shanghai, P. R. China; Department of Radiation Oncology, Fudan University Shanghai Cancer Center, Shanghai, P. R. China; Department of Oncology, Shanghai Medical College, Fudan University, Shanghai, P. R. China; Shanghai Key Laboratory of Radiation Oncology, Shanghai, P. R. China; Department of Radiation Oncology, Fudan University Shanghai Cancer Center, Shanghai, P. R. China; Department of Oncology, Shanghai Medical College, Fudan University, Shanghai, P. R. China; Shanghai Key Laboratory of Radiation Oncology, Shanghai, P. R. China; Department of Radiation Oncology, Fudan University Shanghai Cancer Center, Shanghai, P. R. China; Department of Oncology, Shanghai Medical College, Fudan University, Shanghai, P. R. China; Shanghai Key Laboratory of Radiation Oncology, Shanghai, P. R. China

**Keywords:** metastatic colorectal cancer, SABR, immunotherapy

## Abstract

**Background:**

Currently, the prognosis for metastatic colorectal cancer (mCRC) still remains poor. The management of mCRC has become manifold because of the varied advances in the systemic and topical treatment approaches. For patients with limited number of metastases, radical local therapy plus systemic therapy can be a good choice to achieve long-term tumor control. In this study, we aimed to explore the efficacy and safety of the combination of fruquintinib, tislelizumab, and stereotactic ablative radiotherapy (SABR) in mCRC (RIFLE study).

**Methods:**

RIFLE was designed as a single-center, single-arm, prospective Phase II clinical trial. A total of 68 mCRC patients who have failed the first-line standard treatment will be recruited in the safety run-in phase (*n *=* *6) and the expansion phase (*n *=* *62), respectively. Eligible patients will receive SABR followed by fruquintinib (5 mg, d1–14, once every day) and tislelizumab (200 mg, d1, once every 3 weeks) within 2 weeks from completion of radiation. The expansion phase starts when the safety of the treatment is determined (dose limiting toxicity occur in no more than one-sixth of patients in the run-in phase). The primary end point is the objective response rate. The secondary end points include the disease control rate, duration of response, 3-year progression-free survival rate, 3-year overall survival rate, and toxicity.

**Conclusions:**

The results of this trial will provide a novel insight into SABR in combination with PD-1 antibody and vascular endothelial growth factor receptor inhibitor in the systematic treatment of metastatic colorectal cancer, which is expected to provide new therapeutic strategies and improve the prognosis for mCRC patients.

**Trial registration:**

NCT04948034 (ClinicalTrials.gov).

## Background

Colorectal cancer (CRC) is one of the most common gastrointestinal malignancies in China. The incidence rate was ∼29.51% in 2016, ranking among the top four, with an increasing trend year by year [1]. Approximately 20%–25% of CRC patients are initially diagnosed in the metastatic stage and 25%–50% will develop metastatic disease [2]. About 5%–30% of patients are estimated to present with oligometastatic disease, for whom radical local therapy plus systemic therapy can be used to achieve cure of the tumor or long-term tumor control [3, 4].

Stereotactic ablative radiotherapy (SABR) is a novel radiation treatment method that delivers an intense dose of radiation to the treatment targets with high accuracy. The excellent local control and tolerance profile of SABR have led to its becoming an important modality in cancer treatment. For patients with inoperable early non-small cell lung cancer (NSCLC), SABR has become the standard treatment and brought a great 3-year local control rate of 97.6% at 54 Gy/3 Fx [5]. For oligo-meta patients, SABR could provide substantial survival benefits; for example, a SABR-COMET study showed that SABR led to a higher 5-year overall survival (OS) rate than standard-of-care alone (42.3% vs 17.7%) [6]. Moreover, SABR also exhibited a superb effectiveness when combined with systemic therapy. A Phase II clinical trial (NCT02045446) enrolling patients with limited metastatic NSCLC showed a significant improvement in progression-free survival (PFS) in the SABR-plus-maintenance chemotherapy arm versus the maintenance-chemotherapy-alone arm (9.7 vs 3.5 months), with no difference in toxic effects [7].

Recently, the role of immunotherapy in tumor treatment has been widely investigated, especially for cytotoxic T-lymphocyte associated protein 4 (CTLA-4) and programmed cell death-1 (PD-1) inhibitors. Programmed cell death-ligand 1 (PD-L1) is highly expressed in a variety of malignant tumor tissues, including gastrointestinal tumors. After binding with PD-1 on the surface of T cells, it can significantly inhibit the function of cytotoxic T lymphocytes, induce the production of regulatory T cells, and promote tumor immune escape. PD-1/PD-L1 inhibitors can block the pathway and restore the immune response of T cells to tumors and its efficacy has been widely proven in the treatment of various malignant tumors such as NSCLC and melanoma [8, 9].

Irradiation was shown to sensitize immunotherapy through inducing immunogenic death, remodeling the tumor immune microenvironment and abscopal effect [10–12]. The PACIFIC trial recruited patients with unresectable, stage III NSCLC without disease progression after concurrent chemoradiotherapy and administered durvalumab or not. The results showed that the 4-year OS rates were 49.6% versus 36.3% for durvalumab versus placebo and 4-year PFS rates were 35.3% versus 19.5%, respectively [13]. The PEMBRO-RT study showed that SABR followed by pembrolizumab for advanced NSCLC patients can lead to a higher objective response rate (ORR; 36% vs 18%, *P *=* *0.07), median PFS (6.6 vs 1.9 months, *P *=* *0.19), and median OS (15.9 vs 7.6 months, *P *=* *0.16) at 12 weeks than pembrolizumab alone [14]. Therefore, it seems that the combination of radiotherapy and immunotherapy could achieve better results. From the inspiration of these studies, we attempted to unveil the potential role of SABR combined with immune-checkpoint inhibitors (ICIs) in the treatment of metastatic colorectal cancer (mCRC).

Anti-angiogenic therapy that is focused on inhibiting neovascularization or endothelial cell function has become an indispensable strategy in cancer treatment. The normalization of vascular flow by anti-angiogenesis drugs can reverse hypoxia and the tumor acidic microenvironment, and improve the radiosensitivity of cancer cells [15]. Three months post-SABR, higher efficacy of SABR in lung oligometastases from colon cancer was reported in the cohorts with bevacizumab administration than in those without bevacizumab, with a complete response rate of 64% and 43%, respectively [16]. Therefore, anti-angiogenic therapy combined with SABR is promising. At the same time, anti-angiogenic therapy may have synergistic antitumor effects with ICIs through its immunomodulatory effects [17]. The REGONIVO study of nivolumab plus regorafenib obtained an ORR of 36% and a median PFS of 7.9 months in mCRC patients who progressed after standard therapies [18].

Based on the above theories and practice, we are conducting a Phase II trial of the combination of fruquintinib, tislelizumab, and SABR in mCRC, with the expectation to explore the efficacy and safety of this combination therapy in mCRC patients. The study protocol of this trial, which has the acronym RIFLE, is described in this article.

## Methods and design

### Study design

**Figure 1.**
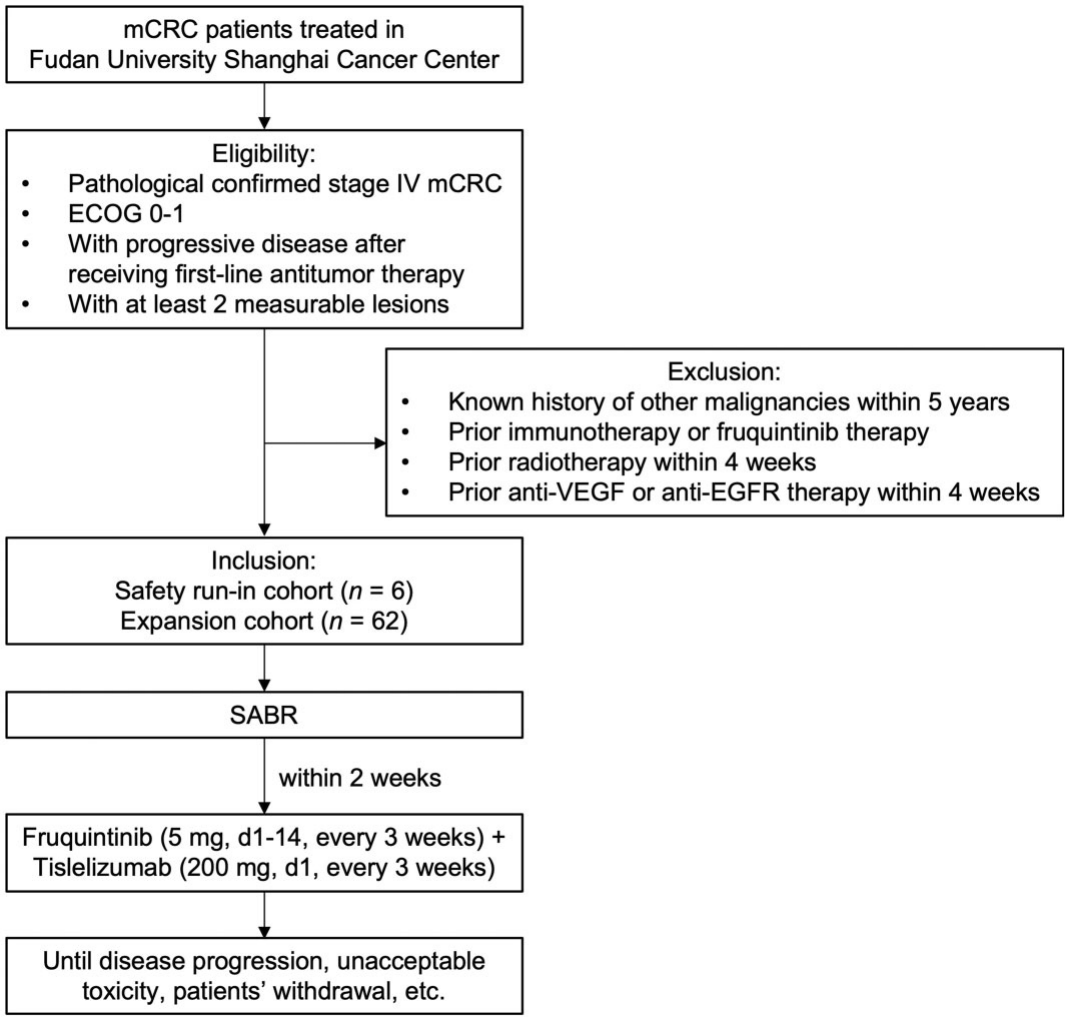
Flow chart of patient selection. mCRC patients who meet the inclusion and exclusion criteria are recruited in this clinical trial. A total of 68 mCRC patients will be enrolled in the safety run-in phase and the expansion phase. Patients will receive SABR followed by fruquintinib and tislelizumab treatment until disease progression, death, toxicity intolerance, consent withdrawal, etc. ECOG = eastern cooperative oncology group, EGFR = epidermal growth factor receptor, mCRC = metastatic colorectal cancer, SABR = stereotactic ablative radiotherapy, VEGF = vascular endothelial growth factor.

The study is a single-center, single-arm, prospective Phase II clinical trial of multisite SABR combined with fruquintinib and tislelizumab in mCRC. Patients who have failed the first-line standard treatment will be recruited and receive multisite SABR followed by fruquintinib plus tislelizumab within 2 weeks until disease progression or intolerable toxicity. This study proposes a two-stage design, with a preliminary run-in cohort to evaluate dose limiting toxicity (DLT) within 21 days after initial drug treatment and a subsequent expansion cohort to ascertain efficacy and safety. The expansion phase can only be launched when the safety run-in phase is completed and the safety of the treatment is determined (DLTs occur in no more than one-sixth of patients). The efficacy of the combination therapy, adverse effects, and long-term prognosis will be analysed. The study algorithm is presented in Figure 1.

### Trial organization, ethics approval, drug supply, and insurance

The trial is initiated by the Department of Radiation Oncology, Fudan University Shanghai Cancer Center (Shanghai, China). The protocol was approved by the Ethics Committee of Fudan University Shanghai Cancer Center (Approval Number: 2011227–6). All patients provide written informed consent before enrollment. Fruquintinib is provided free of charge by Hutchison MediPharma Co., Ltd, which has purchased liability insurance for clinical trial subjects. Tislelizumab is provided through the charity project of BeiGene (Beijing) Co., Ltd.

### Study population

Patients with histologically confirmed mCRC who have previously received systematic treatment for advanced diseases will be recruited. The inclusion criteria and exclusion criteria are as follows; patients who meet the criterion are included in this clinical trial.

### Inclusion criteria

- Age ≥18 years, female and male.
- Pathological confirmed stage IV colorectal cancer (UICC 8th version).
- Eastern Cooperative Oncology Group (ECOG) performance status 0–1.
- Life expectancy of ≥6 months.
- With progressive disease after receiving first-line antitumor therapy (chemotherapeutic agents including fluorouracil, oxaliplatin, and irinotecan) or withdraw from standard treatment before disease progression due to unacceptable toxicity (patients who have failed the second-line standard treatment could be recruited in the safety run-in cohort, but these subjects will not be included in the final statistical analysis).
- With at least two measurable lesions (response evaluation criteria in solid tumors [RECIST] v.1.1).
- Adequate organ function: neutrophils ≥1.5 × 10^9^/L, hemoglobin ≥90 g/L, platelet ≥100 × 10^9^/L, alanine aminotransferase (ALT) ≤2.5 upper limit of normal (ULN), aspartate aminotransferase (AST) ≤2.5 ULN, creatinine ≤1.5 ULN or creatinine clearance ≥50 mL/min, total bilirubin (TBIL) ≤1.5 ULN, APTT ≤1.5 ULN, PT ≤1.5 ULN (the criteria for patients with liver metastasis: platelet ≥80 × 10^9^/L, ALT ≤5 ULN, AST ≤5 ULN, TBIL ≤2.5 ULN).
- Fully informed and willing to provide written informed consent for the trial.

### Exclusion criteria

- Pregnant or lactating women.
- Known history of other malignancies within 5 years except for adequately treated non-melanoma skin cancer, carcinoma *in situ* of cervix, and superficial bladder tumor.
- Prior immunotherapy or fruquintinib therapy.
- Prior radiotherapy within 4 weeks.
- Prior anti-vascular endothelial growth factor (VEGF) or anti-epidermal growth factor receptor (EGFR) therapy within 4 weeks.
- Uncontrolled hypertension: systolic blood pressure (SBP) ≥140 mmHg or diastolic blood pressure (DBP) ≥90 mmHg.
- Uncontrolled malignant pleural effusion, ascites, or pericardial effusion.
- Known history of stroke event or transient ischemic attack within 12 months.
- Known history of arterial thrombosis or deep vein thrombosis within 6 months.
- Known history of clinically significant liver disease, including but not limited to hepatitis B virus (HBV) infection and HBV DNA ≥1 × 10^4^/mL, hepatitis C virus (HCV) infection and HCV RNA ≥1 × 10^3^/mL, and liver cirrhosis.
- Known history of heart disease within 6 months.
- Serious electrolyte abnormalities.
- Urinary protein ≥2+ or 24-hour urine protein ≥1.0 g.
- Gastrointestinal diseases such as gastric or duodenal active ulcers, ulcerative colitis, and unhealed gastrointestinal perforation or fistula.
- Serious mental abnormalities.

### Treatment

Eligible patients will first be treated with multisite SABR for all but one metastatic lesion. Since not all lesions are targeted, we will give priority to symptomatic metastases. The scope of irradiation is the gross tumor area; no preventive irradiation will be given to other areas. The radiation doses of lesions in different sites vary, which should be adjusted according to the tolerance of normal organs at risk, previous literature reports, general condition of patients, and their tolerance to complications. General principles of finite dose for vital organs refer to the report of American Association of Physicists in Medicine Task Group 101 (AAPM-TG101) [19] and the NRG-BR001 trial [20]. Radiotherapy doses for common metastatic sites are referenced as follows: 60 Gy in eight fractions for central lesions of the lung; 50 Gy in five fractions for peripheral lesions of the lung; 48 Gy in eight fractions for the posterior peritoneal lesions; three to eight fractions of 7–10 Gy for the liver lesions. SABR will be administered once daily, five times per week within 3 weeks.

Sequential drug treatment will be initiated within 2 weeks after the final SABR fraction, including tislelizumab 200 mg intravenously d1 and fruquintinib 5 mg orally d1–14 (3 weeks per cycle). Patients will be treated with fruquintinib at a dose of 5 mg daily for the first 14 days of each 21-day cycle. Tislelizumab is administered at a dose of 200 mg every 3 weeks by intravenous infusion. The first infusion should be for ≥60 minutes; if well tolerated, each subsequent infusion should take ≥30 minutes. The duration of tislelizumab is ≤24 months. On the day of the combination treatment, oral fruquintinib is recommended followed by intravenous tislelizumab.

All subjects will receive multisite SABR in combination with fruquintinib and tislelizumab until disease progression, death, patient's request to discontinue study therapy, toxicity intolerance, initiation of new antitumor therapy, pregnancy, serious violation of protocol, investigator's decision to discontinue study therapy based on patient's best interests, or loss of follow-up, whichever comes first.

### DLT

As previously mentioned, this study consists of a safety run-in phase and an expansion phase. The safety run-in phase evaluates DLTs to ensure the safety of the treatment for the subsequent expansion phase. According to the common terminology criteria for adverse event (CTCAE) 5.0 criteria, DLTs are defined for the following adverse events that investigators determine to be associated with fruquintinib and/or tislelizumab and/or multisite SABR within 21 days after initial administration:

- Non-hematological toxicity: grade 3 or above non-hematological toxicity, except for the following conditions:
  - Nausea, vomiting, diarrhea, constipation, and electrolyte disturbance restored to ≤grade 2 within 3 days of supportive treatment;
  - Grade 3 inflammatory response at the site of the tumor;
  - Grade 3 immune-related adverse effects recovered to ≤ grade 2 within 3 days or ≤grade 1 within 2 weeks after treatment;
  - Grade 3 infusion site extravasation;
  - Grade 3 hypertension with SBP ≤ 140 mmHg and DBP ≤ 90 mmHg within 1 week after drug treatment.
- Hematological toxicity:
  - ≥ Grade 3 febrile neutropenia (neutrophil count <1.0 × 10^9/^L, accompanied by a single temperature measurement ≥38.3°C or ≥38°C for 1 hour);
  - Grade 4 thrombocytopenia;
  - Grade 3 thrombocytopenia with severe bleeding;
  - Grade 4 anemia.
- Any other life-threatening toxic reaction.

### Principles of dose adjustment

#### Fruquintinib

If a subject develops a toxic reaction associated with fruquintinib, the adverse effect (AE) will be graded according to CTCAE (version 5.0) and the dose will be adjusted according to the following rules:

- After holding fruquintinib, if AEs can resolve to grade 1 or pretreatment baseline levels within 14 days, treatment can be continued (the 7-day off days in the medication cycle are not counted in this 14-day period).
- If AEs cannot resolve to grade 1 or baseline levels after 14 days of interruption, the subjects will be considered intolerant and fruquintinib will be permanently discontinued.
- Dose adjustment is permitted according to the safety and tolerability of the individual subject, with a reduction of 1 mg/day each time, and the minimum dose level can be lowered to 3 mg/day.
- Once the dose is lowered, it must not be adjusted back to the previous level.

#### Tislelizumab

The dose of tislelizumab is not allowed to be increased or decreased throughout the study. The principles for its interruption or permanent discontinuation are shown in Table 1. AEs associated with PD-1/PD-L1 antibodies are usually called immune-related adverse events (irAEs). For irAE-induced interruption, based on the type and severity, administration can be resumed after treatment. In principle, the use of tislelizumab can be resumed when AEs return to grade 0–1 or baseline levels (in the context of therapeutic glucocorticoid dose of ≤10 mg/day equivalent dose of prednisone) and the ECOG score is 0–1.

### Concomitant medication

#### Prohibited drugs and treatments

- Concurrent treatment with other antitumor therapies, including but not limited to: chemotherapy, radiation therapy, biotherapy, hormone therapy or any other investigational antitumor drug therapy, traditional Chinese medicine with antitumor indications, and immunomodulators are not permitted.
- Simultaneous immunosuppressant and high-dose glucocorticoid therapy (i.e. >10 mg/day of prednisone or equivalent doses of other glucocorticoids, except for drug-related adverse events) are not permitted.
- Immunoglobulins (except for the management of drug-related adverse events), live attenuated vaccine, and autologous hematopoietic stem cell transplantation are not permitted.

#### Permitted drugs and treatments

- Allow the best supportive treatment based on the patient's situation, including but not limited to: antidiarrheals, anti-emetics, opioid or non-opioid analgesics, appetite promoters, and granulocyte and erythrocyte growth factors.
- Topical application (ocular, nasal, intra-articular, inhalational) of steroid hormones is permitted.
- Cortisol is permitted for the treatment of adverse reactions.
- Temporary use of steroids for the prevention and treatment of allergic reactions is permitted.
- Original hormone replacement therapy allowed.
- Bisphosphonates are permitted for bone metastases.
- Medication for pain relief is permitted.

### End points

The primary end point is ORR as assessed by the investigator, per RECIST v.1.1. Non-irradiated and irradiated lesions will be evaluated separately. The secondary end points include the disease control rate (DCR), duration of response (DoR), 3-year PFS rate, 3-year OS rate, and toxicity.

### Assessment

Tumor response assessments will be performed every 6 weeks after the first administration and every 3 months after 1 year until the end of treatment. All the evaluations are done according to RECIST v.1.1.

Safety assessments consist of performing laboratory assessments, measuring vital signs, and monitoring adverse events, including serious adverse events and adverse events of special interest. Adverse events are assessed according to CTCAE v.5.0 and recorded in the patient’s medical record and on the electronic case report form.

### Sample size

This is a single-arm, prospective, Phase II clinical trial. The primary end point is ORR. Sixty-one patients are needed to achieve an ORR of 20%. We assumed that the dropout rate is 10%. Therefore, a total of 68 patients will be needed with a type I error of 5% (both sides) for a statistical power of 80%. The sample size in the safety run-in phase is based on the practical consideration that six participants will be sufficient for preliminary safety evaluation. The sample size in the expansion phase is 62.

### Follow-up

Follow-up will be performed every 3 months from the end of treatment until death, loss of follow-up, withdrawal of informed consent, or the end of the study. The antitumor treatment after disease progression and the date and cause of death will be recorded in detail.

## Discussion

Despite the advances in the systemic treatment of colorectal cancer, prognosis remains dismal due to the high rate of metastases [1, 2]. SABR is a novel radiation method delivering an intense dose of radiation to the targets with a higher dose per fraction and fewer fractions than conventional radiotherapy. Several studies have suggested that SABR can achieve satisfactory tumor control in the local treatment of oligometastases. The 1-year local control rate of SABR was 71%–100% in colorectal cancer patients with fewer than four liver metastasis lesions and tumor diameters of <6 cm [21–24]. The 2-year local control rate was 67%–94% in colorectal cancer patients with fewer than six lung metastasis lesions and tumor diameters of <5 cm [25–27]. In patients with oligometastatic NSCLC that did not progress after front-line systemic therapy, local consolidative therapy with radiotherapy or surgery prolonged PFS (14.2 vs 4.4 months) and OS (41.2 vs 17 months) compared with maintenance therapy or observation [28]. For oligometastatic prostate cancer patients, treatment with SABR improved the median PFS [29]. Thus, we hypothesize that using SABR to eliminate metastatic lesions may help patients to achieve a tumor-free state and long-term survival.

With the emergence of ICIs, PD1/PD-L1 inhibitors have been demonstrated as being effective in mCRC patients with high microsatellite instability (MSI-H) or deficient mismatch repair (dMMR) genes [30, 31]. However, the efficacy of ICIs was limited in ∼90% of advanced colon cancer patients with low microsatellite instability (MSI-L) or proficient mismatch repair (pMMR) genes; the KEYNOTE-016 trial showed that the immune-related ORR was 40% and 0%, respectively, in dMMR and pMMR patients [32]. Although several studies have shown the potential of the immune cell infiltration score [33] and tumor mutation burden [34] to predict immune response in colorectal cancer, how to improve the immunotherapeutic sensitivity of microsatellite stability (MSS) patients is still an urgent problem to be addressed.

Emerging preclinical and clinical evidence has suggested that radiation interacts with the immune system and plays a synergistic role with ICIs. The underlying rationale is that radiation results in the delivery of immunogenic molecules created by the death of tumor cells such as tumor-associated antigens and damage-associated molecular patterns to the immune system and promotes antigen presentation and co-stimulation, thus creating immune responses against previously hidden epitopes that are shared among distant metastases. ICIs can then reverse the immunosuppressive tumor microenvironment and facilitate antitumor immune effects [35]. The “abscopal effect” is classical evidence of the immunomodulatory effect of radiotherapy; it is considered a systemic antitumor immune response induced by local radiotherapy, manifesting as regression of tumors outside of the irradiated field [36]. It was most frequently reported in melanoma treated with ICIs combined with radiation of a single lesion [37, 38]. However, ICIs in combination with single-site radiation do not substantially increase the response rate over that achieved by using ICIs alone. A randomized Phase II trial of nivolumab with single-site SABR versus nivolumab alone in metastatic head and neck squamous cell carcinoma found no significant difference in ORR, OS, or PFS [39]. In a Phase III trial of ipilimumab vs placebo after radiation to a single bone metastatic lesion in patients with castration-resistant prostate cancer, subgroup analysis suggested that patients with lower tumor burden might derive more benefit from the combination therapy [40]. Meanwhile, the overall tumor burden was found to be correlated with the anti–PD-1 response in stage IV melanoma, with a lower tumor burden indicating a better immunotherapy response [41]. These findings suggested to us to maximally reduce the tumor burden to optimize the effects of ICIs. Therefore, in this study, we use comprehensive irradiation of multiple metastases instead of single-site irradiation to enhance the likelihood of obtaining meaningful clinical outcomes. Advantages of multisite irradiation include better antigen presentation, improved immune access, lower tumor burden, and reduced immunosuppressive effects of bulky lesions [35]. A Phase I study of pembrolizumab with multisite stereotactic body radiation therapy in patients with advanced solid tumors obtained a decent median PFS of 3.1 months [42]. As for MSS mCRC patients, Parikh *et al*. [43] reported that ipilimumab and nivolumab with multisite SABR obtain a DCR of 37% and a median PFS of 2.5 months, providing evidence of combining multisite radiation with immune-checkpoint blockade in immunotherapy-resistant cancers.

Numerous preclinical and clinical studies have focused on the combination of anti-angiogenic agents and ICIs in the past decades. The rationale for the combination relies on anti-angiogenic agents promoting antigen presentation, activating cytotoxic CD8+ T cells, and promoting the infiltration and migration of lymphocytes. ICIs can enhance the antitumor effects of anti-angiogenic agents by relieving immunosuppression [44]. The REGONIVO trial combined regorafenib with nivolumab in patients with gastric and colorectal cancer who had received at least two previous lines of chemotherapy [18]. It achieved an ORR of 40% and demonstrated encouraging antitumor activity of the combined therapy [18], although the results in the North American population were not consistent with those in the Japanese population [45]. The LEAP-005 trial of lenvatinib plus pembrolizumab in patients with previously treated advanced non–MSI-H CRC also obtained a promising ORR of 21.9% and DCR of 47% [46]. Fruquintinib is a China-made anti-angiogenic drug that is approved for third-line therapy in mCRC. In preclinical studies, a combination of fruquintinib and PD-1 blockade exhibited stronger inhibition of tumor growth in both MSS and MSI CRC models than either single agent alone [47]. Fruquintinib even showed better efficacy than regorafenib when combined with PD-1 inhibitors as a third-line or above-posterior-line therapy in patients with mCRC in a retrospective study [48] in which the DCR and the median PFS were higher in the fruquintinib-plus-PD-1 inhibitor group than in the regorafenib-plus-PD-1 inhibitor group (DCR: 89.3% vs 56.5%; PFS: 6.4 vs 3.9 months). In a multicenter prospective study of fruquintinib plus sintilimab in mCRC, the ORR was 27.3%, the DCR was 95.5%, and the median PFS was 6.9 months in the 5-mg intermittent cohort [49]. Fruquintinib also showed encouraging efficacy when combined with geptanolimab in mCRC patients (80% MSS, 6.7% MSI-H, and 13.3% unknown) who had failed one or two standard therapies, with a Phase Ib trial achieving an ORR of 26.7%, DCR of 80%, and median PFS of 7.33 months [50]. In our study, we adopt fruquintinib and tislelizumab to investigate whether this combination could obtain encouraging outcomes that are similar to those reported in the previous studies in mCRC patients.

Furthermore, radiotherapy plays a significant role in the context of anti-angiogenic therapy plus ICIs. In addition to the previously described synergies between immunotherapy and radiotherapy or anti-angiogenic agents, radiotherapy also has an effect on tumor vasculature. Single high-dose irradiation induces endothelial cell apoptosis and senescence, causing vessel regression and collapse. This eventually results in tissue hypoxia, which leads to a vascular rebound effect by growth factor-induced angiogenesis. Fractionated low-dose irradiation induces an increased expression of angiostimulatory growth factors such as VEGF and basic fibroblast growth factors. This promotes different endothelial cell functions that result in vascular growth induction and enhance tissue perfusion. Both the vascular rebound effect and the vascular growth induction provide opportunities for intervention of anti-angiogenic agents [51]. Since the combination of regorafenib with nivolumab achieved different results in different populations [18, 45], radiotherapy is taken into consideration to modify the tumor vasculature and immune microenvironment. Thus far, there have been few clinical trials combining radiotherapy, anti-angiogenic therapy, and immunotherapy together. The preliminary results of a Phase I/II study of regorafenib and pembrolizumab in refractory MSS CRC showed that the median PFS for those who received radiotherapy previously was 4.4 months, while for those who had not received radiotherapy, the median PFS was only 1.8 months [52]. A retrospective study investigated the clinical outcomes and safety of PD-1/PD-L1 inhibitors combined with palliative radiotherapy and anti-angiogenic therapy in advanced Barcelona clinic liver cancer (BCLC) stage C hepatocellular carcinoma [53]. The results showed that the ORR, median PFS, and median OS were 40.0%, 4.7 months, and 21.2 months, respectively, with no unexpected adverse events. The ORR was promising compared with previous studies of ICIs plus anti-angiogenic agents including BCLC stage A–C patients obtaining an ORR of 22%–36% [54–56]. Another Phase I study also obtained encouraging results of pembrolizumab, hypofractionated stereotactic irradiation, and bevacizumab in patients with recurrent high-grade gliomas [57]. The ORRs were 83% and 62%, and the median OSs were 13.45 and 9.3 months, respectively, in the bevacizumab-naïve cohort and the bevacizumab-resistant cohort [57]. Collectively, the findings described above showed an encouraging efficacy of the triple combination therapy. Therefore, we propose that it would be meaningful to examine the efficacy of anti-angiogenic drugs and ICIs following SABR in advanced colorectal cancer patients.

In summary, RIFLE is a single-arm, prospective Phase II clinical trial to investigate the efficacy and safety of the combination of fruquintinib, tislelizumab, and SABR in mCRC patients who have failed the first-line standard treatment. The results of this study will help to enhance the current understanding of the combination of these three strategies and improve clinical practice in the systematic treatment of metastatic colorectal cancer.

## Authors' Contributions

F.X. and Z.Z. built the conception and designed the study. F.X., Y.J.C., K.W., and Z.Y.Z. made substantial contributions to the organization of this trial. F.X., Z.Z., J.F.W., L.J.S., H.Z., and Y.W. were responsible for patient recruitment. F.X., Y.J.C., and Z.Y.Z. contributed to the evaluation of tumor responses. F.X., X.H., and J.Z.W. carried out the power calculation and data analysis. K.W. wrote the first draft of the manuscript, and all authors read and approved the final manuscript.

## Figures and Tables

**Table 1.** Tislelizumab administration adjustment scheme

irAE	Severity	Adjustment
Pneumonia	G2 pneumonia	Hold
	Recurrent G2 pneumonia, G3–4 pneumonia	Permanently discontinue
Diarrhea/colitis	G2–3 diarrhea/colitis	Hold
	G4 diarrhea/colitis	Permanently discontinue
Hepatitis	G2 hepatitis, 3 ULN ≤ ALT/AST ≤ 5 ULN or 1.5 ULN ≤ TBIL ≤ 3 ULN	Hold
	G3–4 hepatitis, ALT/AST ≥ 5 ULN or TBIL ≥ 3 ULN	Permanently discontinue
Renal insufficiency	G2–3 elevated serum creatinine	Hold
	G4 elevated serum creatinine	Permanently discontinue
Endocrine adverse events	Clinical G2–3 hypothyroidism/thyrotoxicosis G2–3 hypophysitis G2 adrenal insufficiency G3 hyperglycemia or type I diabetes mellitus	Hold
	G4 hypothyroidism/thyrotoxicosis G4 hypophysitis G4 adrenal insufficiency G4 hyperglycemia or type I diabetes mellitus	Permanently discontinue
Dermal toxicity	G3 dermatitis	Hold
	G4 dermatitis, Stevens–Johnson syndrome, or toxic epidermal necrolysis	Permanently discontinue
Thrombocytopenia	G3 thrombocytopenia	Hold
Other irAEs	G3–4 elevation in amylase/lipase G2–3 pancreatitis G2 myocarditis Other G2–3 irAEs occurred for the first time	Hold
	G4 pancreatitis or recurrent pancreatitis of any grade G3–4 myocarditis G3–4 encephalitis Other G4 irAEs occurred for the first time	Permanently discontinue
Recurrent or persistent AE	Recurrent G3–4 AE (except for endocrine disease) G2–3 AEs not return to grade 0–1 or baseline levels within 12 weeks after the last dose (except for endocrine disease) Glucocorticoids not decrease to a prednisone equivalent dose of <10 mg/day within 12 weeks after the last dose	Permanently discontinue
Infusion-related reactions	G2 infusion-related reactions	Slow the rate of infusion or hold immunotherapy
	G3–4 infusion-related reactions	Permanently discontinue

The above adverse event severity evaluation is based on NCI-CTCAE v.5. irAE = immune-related adverse event, G1/2/3/4 = grade 1/2/3/4, ULN = upper limit of normal, ALT = alanine aminotransferase, AST = aspartate aminotransferase, TBIL = total bilirubin, AE = adverse event.
